# Developing a list of Alert Alien Species in South Korea

**DOI:** 10.3897/BDJ.12.e125517

**Published:** 2024-08-14

**Authors:** Aram Jo, Jihee Kim, Jounghun Park, Yunjeong Cho, Su-gon Park, Dong Eon Kim

**Affiliations:** 1 Invasive Alien Species Team, National Institute of Ecology, Seocheon 33657, Republic of Korea Invasive Alien Species Team, National Institute of Ecology Seocheon 33657 Republic of Korea; 2 Department of Biological Science, Kongju National University, Kongju 32588, Republic of Korea Department of Biological Science, Kongju National University Kongju 32588 Republic of Korea; 3 Division of Life Sciences, Korea Polar Research Institute, Incheon 21990, Republic of Korea Division of Life Sciences, Korea Polar Research Institute Incheon 21990 Republic of Korea; 4 Department of Life Sciences, Yeungnam University, Gyeongsan, Republic of Korea Department of Life Sciences, Yeungnam University Gyeongsan Republic of Korea; 5 Research Policy Planning Team, National Institute of Ecology, Seocheon 33657, Republic of Korea Research Policy Planning Team, National Institute of Ecology Seocheon 33657 Republic of Korea

**Keywords:** Alert Alien Species, biological invasions, alien species management, precautionary approach

## Abstract

Along with transportation development, climate change and socio-economic changes, invasive alien species (IAS) are causing a significant decline in biodiversity around the world. Internationally, policies for pre-invasion management of IAS are being emphasised to minimise damage from biological invasions. In South Korea, through the 2^nd^ Alien Species Management Plan (2019–2023), IAS that are not yet present in the country but are likely to be introduced are designated as Alert Alien Species (AAS). In this study, the overall process of AAS designation is summarised and improvements to the current system are presented. To select AAS, an invasive alien species database (IASD) of 8,456 species was built by integrating the IAS lists from many countries. Amongst them, 1,534 species, included in IASD at genus, family and order level, were excluded and 3,298 species confirmed to have been introduced to South Korea were excluded from the AAS candidate species. After the creation and review of species profiles by experts, 150 species were finally designated as AAS in 2023. The AAS discovery process needs to reflect international trends of IAS and be continuously supplemented through policy research of other countries. In addition, the IAS management system in South Korea, in which various ministries play their own roles with sufficient data sharing, should be systematically linked from introduction to control of IAS.

## Introduction

As part of the Earth’s ecosystem, the conservation of biodiversity is no longer a discretionary action, but an imperative responsibility for our survival. To fulfil this global responsibility, representatives and experts from 196 parties convened in Montreal, Canada in December 2022 to adopt the 2030 Global Biodiversity Framework at the 15^th^ Conference of the Parties to the Convention on Biological Diversity (CBD 2022). Amongst the 23 targets that countries must comply with, the sixth, “reduce the introduction of invasive alien species (IAS) by 50% and minimise their impact,” suggests that IAS are a major cause of biodiversity loss (Simberloff et al. 2013, Chapman et al. 2016). The economic damage caused by IAS globally exceeds USD 1.28 trillion (Diagne et al. 2021). A previous study predicted the risk of invasion, based on the introduction and establishment data of IAS; they found that 17% of the world’s inland areas are highly vulnerable to invasion, with most regions in South Korea exhibiting a high level of vulnerability (Early et al. 2016). The study confirmed that the number of alien species that have entered South Korea has experienced a two-fold increase, increasing from 1,109 in 2011 to 2,160 in 2018 and has steadily continued to increase, reaching 2,653 species in 2021 (National Institute of Ecology 2021, Hong et al. 2022).

The international consensus on the most economical and environmentally desirable method to minimise the impact of IAS is prevention before invasion (Simberloff et al. 2013). Since not all alien species are harmful and it is impossible to block all pathways of introduction, it is imperative to proactively identify species with potential impact, before introduction and to adopt management strategies that specifically target them (Faulkner et al. 2014, Singh et al. 2015, McGeoch et al. 2016). The guiding principles for alien species management from the Subsidiary Body on Scientific, Technical and Technological Advice (SBSTTA) emphasise a precautionary approach, prioritising prevention before any management actions (CBD 2000). According to previous research results, after establishing strict biosecurity policies on the introduction of alien species since the 19^th^ century, New Zealand had almost no invasion of alien mammals compared to Europe and Australia’s quarantine programme for potential invasive plants has prevented economic losses of USD 1.67 billion over 50 years (Simberloff et al. 2013).

The primary objective of South Korea’s First Alien Species Management Plan (2014–2018) was to address the eradication of introduced alien species causing ecological and economic damage; however, it failed to adequately emphasise the crucial aspect of managing the introduction of these species. To resolve this, the Second Alien Species Management Plan (2019–2023) included measures to substantially strengthen the pre-introduction management of alien species (Ministry of Environment 2019). One of the main issues is to strengthen the prevention of introduction of non-introduced species suspected of being invasive and the Ministry of Environment designates species that could harm the ecosystem if introduced to South Korea as Alert Alien Species (AAS) through the Act on the Conservation and Use of Biological Diversity (hereafter, the “Biodiversity Act”). Two hundred species were first designated as AAS in 2019 and, as of October 2023, 712 species have been designated and six species have been delisted, resulting in a total of 706 species (Ministry of Environment 2023). Species designated as AAS undergo an ecological risk assessment (ERA) when imported or brought into the country for the first time to decide on their authorisation. For this, importers must submit documents to the head of the local environmental office; this documentation comprises a species certification issued by the exporting country, a usage plan and major expected routes of exposure to the ecosystem. Based on the ERA results, species are either reclassified as legal management species, with import, distribution, breeding, legally prohibited or removed from the AAS list if they are deemed to have insufficient risk to warrant legal restrictions. Additionally, AAS that enter through unintentional or illegal routes and are discovered in natural ecosystems must also undergo an ERA to assess their legal management status (Kim 2018, Son et al. 2021).

Previous research on AAS includes studies analysing the risk and damage cases of AAS and predicting the possibility of AAS introduction through various pathways, including trade volume (Kim 2018, Son et al. 2021, Jo et al. 2022). However, there has been a lack of research on the process of building a list of alien species, requiring immediate management as a priority in South Korea. Therefore, the aim of this study was to present the status of non-introduced alien species and prepare improvement measures by systematising the process for discovering AAS.

## Material and methods

### Integration for Invasive Alien Species Database (IASD)

**Figure 1.**
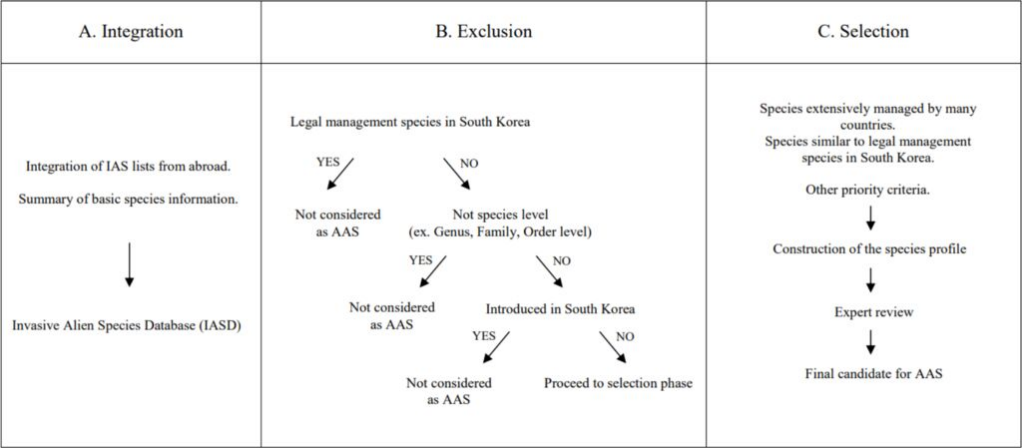
Workflows for developing a list of Alert Alien Species (AAS).

The first step in the process for discovering AAS is the integration phase, where lists of IAS reported by other countries were organised to construct the IASD (Fig. 1A). The collection of IAS lists referenced global and regional IAS databases, national reports, research papers and laws from neighbouring countries, such as Japan and China, as well as major trading nations, such as the United States and EU countries. The IASD includes basic information, such as taxonomic group, family, scientific name, common name, managing countries and domestic introduction records and whether it is already designated as a legal management species in South Korea. The database covers eight taxonomic groups, excluding marine species, microorganisms, some insects and arthropods that are too small for identification with the naked eye. It includes mammals, birds, fish, molluscs, arthropods (including spiders and insects), amphibians, reptiles and plants. For plants, it was limited to relatively easily identifiable vascular plants, excluding algae and mosses. For fish, the class Actinopterygii, which includes a large number of freshwater bony fish species, was preferentially included. AAS has been designated every year since 2019 and sources of IASD are added, supplemented and updated every year. In this paper, we summarise the process of building the most recently updated IASD and designating additional AAS list specifically for the year 2023.

### Exclusion of introduced alien species

The second phase was performed to exclude species not suitable for AAS from the IASD constructed through data research (Fig. 1B). In this exclusion phase, species already managed for their import and entry under the Biodiversity Act were excluded. In addition, since AAS are designated at the species level, species designated at the genus, family or order level in other countries were excluded. To prevent the introduction of alien species, it is appropriate to manage at the genus, family and order level for the precautionary approach. The main aim of the current AAS designation system blocks the import of species that meet five criteria before identifying the specific risks of the species and screens the risks before they are introduced into the country. Therefore, if AAS is designated at the genus, family and order level, a risk assessment must be performed for all applicable species and, due to the difficulty of increasing import restrictions, it is currently designated at the species level. Lastly, species already confirmed to be introduced in South Korea were excluded. The determination of whether a species had been introduced in South Korea was based on the utilisation of resources such as the National list of species of Korea (National Institute of Biological Resources 2021), Checklist of Vascular Plants in Korea (Korea National Arboretum 2022) and Guide for Nationwide Survey of Non-native Species in Korea (National Institute of Ecology 2021). Additionally, species with records of personal breeding or commercial trading in South Korea discovered through internet searches were also excluded.

### Selection amongst candidates for AAS

In the selection phase, the candidates that remained underwent screening to ascertain the appropriate prioritisation of AAS for each taxonomic group (Fig. 1C). Although managing all AAS candidate species is most effective on a precautionary level, it is efficient to select and designate specific species that will be given priority every year, taking into account the budget and manpower. Therefore, it was determined that the more countries or regions that manage invasive alien species and the more ecologically and genetically similar they are to legally managed species managed under the Biological Diversity Act, the more priority they should be designated as AAS. Next, species profiles were created by experts in each taxonomic group. A species profile requires compiling data on the morphological and ecological characteristics, risks and damage cases, likelihood of introduction and distribution outside South Korea; thus, experts were selected, based on their research experience in biology, ecology and natural sciences or involvement in alien species-related tasks. The collected species profiles were reviewed by a newly-formed selection committee of at least five experts per taxonomic group, considering their empirical knowledge to determine whether the species fulfilled the criteria for designation as AAS; subsequently, the final candidates were selected. To reduce potential bias due to experts’ subjective judgements, a comprehensive reflection of multiple expert opinions was incorporated. The criteria for designating AAS are as follows:

1. Species internationally recognised as invasive such as 100 of the World’s Worst Invasive Alien Species by the International Union for Conservation of Nature (IUCN) or regulated by law in neighbouring countries (such as China and Japan) and major trading partners (including the United States and EU countries).
2. Species known to have caused social or ecological damage.
3. Species that are genetically or ecologically similar to legal management species.
4. Species with a high probability of survival and reproduction because their original habitats are similar to domestic environments.
5. Species known to impact human health through diseases or toxicity. The final candidate species that meets one or more of the above criteria were finally notified as AAS by the Ministry of Environment after consultation with relevant organisations.

## Results

### Integration for Invasive Alien Species Database (IASD)

In the research to discover AAS, we collected various references, such as websites, books and laws from global, regional and national scopes and ultimately compiled 50 references (Table 1, Suppl. material 1). Data sources for the global IAS list included the International Union for Conservation of Nature (IUCN) 100 of the World’s Worst Invasive Alien Species and Invasive Alien Species: Observations and Issues from Around the World. The IUCN’s IAS list is globally recognised as the most authoritative list of species reported for causing significant harm to biodiversity and human activities, representing major cases of biological invasion (Lowe et al. 2000). Data on IAS in Southeast Asia were mostly compiled from Invasive Alien Species: Observations and Issues from Around the World (Pullaiah and Ielmini 2021). This information includes data related to alien species from all continents, including Africa, Asia, the Pacific Region, Europe, the Americas and the Caribbean. The regional and national IAS lists were compiled by combining various references. For Japan, the list of alien species potentially harmful to the ecosystem announced by the Ministry of the Environment was utilised. For China, lists of IAS announced by the Ministry of Ecology and Environment and the Ministry of Agriculture and Rural Affairs, as well as those provided by the Research Center for the Prevention and Control of Invasive Organisms, were compiled. Additionally, lists of import plant quarantine pests used by the Chinese Customs and research papers describing IAS of China were also compiled. In the case of Hong Kong, the species listed in the Biodiversity Strategy and Action Plan (2016-2021), as well as plants whose import is legally controlled by the Plant (Importation and Pest Control) Ordinance, were utilised. For the United States, lists of injurious species announced under the Lacey Act and lists of noxious weeds were compiled. Additionally, lists of species that have undergone ecological risk screening by the U.S. Fish & Wildlife Service and lists of alien species provided by the invasive.org ecosystem health centre were compiled. invasive.org is a joint project by the University of Georgia, including the USDA Animal and Plant Health Inspection Service, USDA Forest Service, USDA Identification Technology Program and USDA National Institute of Food and Agriculture, compiling lists and statuses of invasive and exotic species by state. In Canada, IAS management is regulated jointly by federal and provincial laws, as described by the Invasive Species Centre. The lists of IAS managed at the national level were compiled from the Aquatic Invasive Species Regulations and the Plant Protection Regulations and lists from the Invasive Species Act enacted in Ontario were also included at a provincial level. For Australia and New Zealand, due to their unique environments and high biodiversity, the management of alien species is very detailed and strict. Governments, as well as states, territories and regional councils, implement their own alien species management plans. Therefore, the lists of IAS in Australia were compiled from the Australian Weeds/Pest Animal Strategy 2017 to 2027, the Exotic Environmental Pest List (EEPL) and lists of invasive species announced by the Department of Climate Change, Energy, the Environment and Water. In addition, lists of invasive species managed by each state and territory in Australia were also included. Similarly, in New Zealand, various laws related to alien species, such as the Biosecurity Act, HSNO Act and Wildlife Act exist, with highly diverse categories for classifying alien species, such as Unwanted, Notifiable, New Organism and Prohibited. Regional councils manage and control pests in their areas according to their own pest management plans, so New Zealand’s lists of IAS were compiled from the Ministry for Primary Industries’ list of pests of concern, the Department of Conservation’s consolidated list of environmental weeds and the list of unprotected wildlife under the Wildlife Act. Lists of alien species managed at the regional level in New Zealand were utilised from the information centre (bionet.nz) supporting the government’s Biosecurity 2025 plan. In Southeast Asia, the list was compiled, based on lists published by government ministries, related papers and reports. In Europe, lists were compiled from the priority pest list for the plant health and the Union List, which is the core of the IAS laws adhered to by EU Member States. In compliance with EU regulations, additional laws and lists of IAS according to the ecosystem characteristics of each country were also included in the Europe’s IAS list. During the process of compiling the lists from each source, synonyms were organised and, ultimately, IASD containing a total of 8,456 species was constructed to discover AAS candidates in 2023 (Table 2). Plants accounted for more than half with 4,697 species, followed by arthropods, including insects and spiders, which comprised 1,342 species.

### Exclusion of introduced alien species

Amongst the 8,456 species included in the IASD, a total of 1,534 species were excluded after verifying species already managed under the Biodiversity Act and those included at the genus, family or order levels (Table 2A). Plants were the most excluded group with 641 species, followed by fish with 260 species and more than 100 species of mammals, amphibians, reptiles and arthropods were also excluded. The taxonomic group with the highest percentage of excluded species was amphibians, with 70.3% excluded, followed by reptiles (35.7%) and fish (35.1%). Out of the remaining 6,922 species, 3,298 were further excluded after identifying those already introduced to South Korea (Table 2B). Plants were excluded the most with 1,980 species and more than 200 species were excluded from all other taxonomic groups, except amphibians and molluscs. The taxonomic group with the largest percentage of excluded species was reptiles, with 85.1% excluded, followed by mammals (78.2%) and birds (54.2%). After the exclusion phase, 3,624 candidate species remained, with plants being the most included at 2,076 species, followed by arthropods, including insects and spiders at 894 species, comprising 81.9% of the total.

### Selection amongst candidates for AAS

For efficient AAS management, we screened the species with the highest management priority for each taxonomic group and a total of 344 species were selected for profile creation (Table 2C). Arthropods had the highest representation with a total of 112 species, followed by plants and fish with 73 and 40 species, respectively. After thorough evaluation of the species profiles and AAS designation criteria by the selection committee of experts for each taxonomic group, a total of 150 species were selected as AAS (Table 2D). Arthropods were identified as having the most abundant species, with a total of 56, whereas plants and molluscs ranked second and third with 27 and 18 species, respectively. Since the first designation of 200 species in 2019, 712 species have been designated as AAS over a span of 5 years until 2023, with six species delisted (Table 3, Suppl. material 2). Depending on the results of the risk assessment, six species were either reclassified as legal management species or no longer managed by law. Amongst the eight taxonomic groups and 706 species included in AAS, plants were the most abundant with a total of 241 species, constituting approximately 34.1% of the total, followed by fish at 131 species, representing 18.5%.

## Discussion

In South Korea, the Ministry of Environment has been designating and restricting the import and entry of species that could harm the ecosystem as AAS since 2019 to proactively manage non-introduced alien species. However, in the process of selecting AAS with high management priority amongst many alien species, in order to exclude species that have been introduced into South Korea, even if they are not found in the natural ecosystem after being intentionally introduced, such as pets and cultivated plants, they are sometimes excluded from the AAS candidates. Therefore, in order to prevent early-stage invasive species from being overlooked, it is necessary to consider including species that have begun to be introduced as candidate species (Carboneras et al. 2018).

The fundamental difficulty encountered in the process of discovering AAS was the lack of accurate understanding of alien species management systems of other countries. Thus, there may be references included in the IASD with questionable validity or missing due to lack of information, necessitating ongoing improvements through continuous policy research on the global trends and systems of alien species management. In addition, IAS management system in South Korea also needs to be effectively linked with related government ministries. There were different lists and laws of alien species followed by various ministries, such as the Animal and Plant Quarantine Agency, Rural Development Administration, Korea Forest Service, Ministry of Oceans and Fisheries and the Ministry of Environment. While effective and specific management can be achieved in this way, an integrated IAS management system for data sharing is crucial for future collaborative efforts (Hao and Ma 2023). Establishing centres for integrated management of IAS, such as the invasive.org project in the United States and Canada’s Invasive Species Centre, is necessary to systematically manage alien species data from introduction to management.

An additional difficulty in developing lists for AAS was that the selection process relies substantially on the subjective opinions of experts rather than indicators and scores. Selecting potentially invasive species from many alien species is a complex task requiring significant consideration (Vilà et al. 2010, Singh et al. 2015, Roy et al. 2019). For non-introduced alien species, predicting the actual ecological impact upon introduction in South Korea is challenging due to climate change and various conditions, often requiring judgement based on possibilities. As these impacts can be identified through continuous research and monitoring, many policies of other countries, which must consider multiple taxa and species at once, utilised various screening tools including designation criteria and expert consultations (Vilà et al. 2010, Simberloff et al. 2013, Faulkner et al. 2014, Singh et al. 2015). Moreover, as in the alien species management principles presented in SUBSTTA, the lack of scientific certainty about the impact of IAS should not be a limitation of IAS prevention policies, so it is important to obtain expert opinions on the possibility of invasiveness (CBD 2000). To prioritise target species for future eradication and control, research is needed on objective and effective AAS identification systems and specific risk assessments of the possibility of introduction, establishment and impact on ecosystems of designated AAS.

The list of legally announced AAS must be flexible and responsive to rapidly adapt to the spread and introduction of alien species (Carboneras et al. 2018). Future research on objective and effective system of discovering AAS and specific risk assessment of designated AAS for prioritising target species for eradication is necessary. The systematic AAS selection process described in this study is anticipated to help identify improvements in current management strategies and provide valuable insights for the precautionary approach for non-introduced alien species.

## Supplementary Material

0D3092CD-D4A4-5E08-9DD4-C3689A9030C2 10.3897/BDJ.12.e125517.suppl1 Supplementary material 1 Sources of invasive alien species list Data type List of reference in Table 1 File: oo_1023768.xlsx https://binary.pensoft.net/file/1023768 Aram Jo

F36070A7-25E3-57F3-8936-1ADF78EFE2A3 10.3897/BDJ.12.e125517.suppl2 Supplementary material 2 List of Alert Alien Species (AAS) in South Korea Data type List of species File: oo_1025805.xlsx https://binary.pensoft.net/file/1025805 National Institute of Ecology

## Figures and Tables

**Table 1.** List of references of invasive alien species (IAS).

**Scope**	**Sources of invasive alien species list**	
Global	IUCN 100 of the World’s worst invasive alien species	
	Invasive Alien Species: Observations and Issues from Around the World	
Japan	List of regulated living organisms under the Invasive Alien Species Act	
China	List of Invasive Alien Species in China’s Natural Ecosystem	
	Key management list of invasive alien species	
	Database of Invasive Alien Species in China	
	Invasive alien plants in China: An update	
	An inventory of invasive alien species in China	
	List of Imported Plant Quarantine Pests of the People’s Republic of China	
Hong Kong	Hong Kong Biodiversity Strategy and Action Plan (2016–2021)	
	Plant (Importation and Pest Control) Ordinance	
USA	Invasive and Exotic Species of North America	
	Designation of noxious weeds	
	Ecological risk screening summary List	
	Species Currently Listed as Injurious Wildlife under the Lacey Act	
Canada	List of pests regulated by Canada	
	Aquatic invasive species	
	Invasive species in Ontario	
Australia	Invasive species of Australia	
	The National Priority List of Exotic Environmental Pests, Weeds and Diseases	
	Government pest animal legislation, strategies and plans	
New Zealand	Pests of concern to New Zealand	
	Consolidated list of environmental weeds in New Zealand	
	Unprotected wildlife	
	Pest management plans	
Southeast Asia	Invasive Alien Species in South-Southeast Asia	
	Sri Lanka	Regulated Weed List NPPO - Sri Lanka
		Invasive Alien Species in Sri Lanka
	Philippines	Invasive Alien Species in the Philippines
	Nepal	Invasive alien plant species (IAPS) in Nepal
	India	List of Invasive Alien plant species in India
		Invasive Alien Species of India
	Indonesia	A Guide Book to Invasive Plant Species in Indonesia
		List of fish species that are harmful and/or detrimental
	Malaysia	National action plan on invasive alien species 2021-2025
Europe	List of Invasive Alien Species of Union concern (Union List)	
	List of priority pests	
	Finland	Nationally significant harmful alien species
	Slovakia	List of invasive animal species and invasive plant species
	Portugal	National List of Invasive Species (LNEI)
	Poland	List of Invasive alien species (IGOs) posing a threat to the Union/Poland
	Spain	Spanish Catalogue of Invasive Alien Species
	Denmark	Invasive non-native species on the EU list and on a national list
	Lithuania	List of invasive species in Lithuania
	Estonia	Natural list of invasive alien species
	Bulgaria	Invasive Alien Plant Species in Bulgaria
NOBANIS	Invasive Alien Species Pathway Analysis and Horizon Scanning for Countries in Northern Europe	
	The NOBANIS fact sheets of the worst invasive alien species	
UK	List of GB non-native species	
	Regulated invasive alien species of the counties of the United Kingdom	

**Table 2.** Number of species by taxonomic group throughout the process of discovering AAS candidates. The percentages in bold in remaining species indicate the proportion of species within each taxonomic group out of the total number of species and the percentages in excluded species represent the proportion of species excluded from the previous stage. **A.** Species included in IASD at a genus, family and order level and those already managed under the Biodiversity Act. **B.** Species confirmed to be introduced in South Korea. **C.** Species considered to require further risk investigation. **D.** Candidate species for AAS.

**Taxon**	**IASD**	**Exclusion**				**Selection**			
		**A. Classification**, **under the Biodiversity Act**		**B. Introduced species**		**C. Species profile**		**D. Alert Alien Species**	
		**Excluded species**	**Remaining species**	**Excluded species**	**Remaining species**	**Excluded species**	**Remaining species**	**Excluded species**	**Remaining species**
Mammalia	476 **(5.6%)**	145 (30.5%)	331 **(4.8%)**	259 (78.2%)	72 **(2.0%)**	46 (63.9%)	26 **(7.6%)**	16 (61.5%)	10 **(6.7%)**
Aves	463 **(5.5%)**	39 (8.4%)	424 **(6.1%)**	230 (54.2%)	194 **(5.4%)**	165 (85.1%)	29 **(8.4%)**	24 (82.8%)	5 **(3.3%)**
Fish	741 **(8.8%)**	260 (35.1%)	481 **(6.9%)**	248 (51.6%)	233 **(6.4%)**	193 (82.8%)	40 **(11.6%)**	25 (62.5%)	15 **(10.0%)**
Amphibian	192 **(2.3%)**	135 (70.3%)	57 **(0.8%)**	23 (40.4%)	34 **(0.9%)**	10 (29.4%)	24 **(7.0%)**	9 (37.5%)	15 **(10.0%)**
Reptile	375 **(4.4%)**	134 (35.7%)	241 **(3.5%)**	205 (85.1%)	36 **(1.0%)**	23 (63.9%)	13 **(3.8%)**	9 (69.2%)	4 **(2.7%)**
Mollusca	170 **(2.0%)**	35 (20.6%)	135 **(2.0%)**	50 (37.0%)	85 **(2.3%)**	58 (68.2%)	27 **(7.8%)**	9 (33.3%)	18 **(12.0%)**
Arthropoda	1,342 **(15.9%)**	145 (10.8%)	1,197 **(17.3%)**	303 (25.3%)	894 **(24.6%)**	782 (87.5%)	112 **(32.6%)**	56 (50.0%)	56 **(37.3%)**
Plant	4,697 **(55.5%)**	641 (13.6%)	4,056 **(58.6%)**	1,980 (48.8%)	2,076 **(57.3%)**	2,003 (96.5%)	73 **(21.2%)**	46 (63.0%)	27 **(18.0%)**
Total	8,456 (100%)	1,534	6,922 **(100%)**	3,298	3,624 **(100%)**	3,280	344 **(100%)**	194	150 **(100%)**

**Table 3.** Number and proportion of taxon of AAS by designation year. * After designating 200 species in 2019, four species (*Pygocentrusnattereri* (Piranha), *Salmosalar* (Atlantic Salmon), *Xenopuslaevis* (African Clawed Frog) and *Anoplolepisgracilipes* (Yellow Crazy Ant)) were reclassified as legal management species and *Paramisgurnusdabryanus* (Chinese Loach) were no longer managed by law. ** After designating 160 species in 2022, *Solenopsisgeminata* (Tropical Fire Ant) was reclassified as a legal management species due to high risk.

**Taxon**	**2019**	**2020**	**2021**	**2022**	**2023**	**Total number of ASS**	**Proportion (%)**
Mammalia	10	15	10	11	9	55	7.8
Aves	7	0	4	10	6	27	3.8
Fish	*58	23	16	21	13	131	18.5
Amphibian	*22	5	5	12	13	57	8.1
Reptile	14	8	11	8	4	45	6.4
Mollusca	1	0	17	0	18	36	5.1
Arthropoda	*33	0	24	**1	56	114	16.1
Plant	50	49	15	96	31	241	34.1
Total	*195	100	102	**159	150	706	100.0
